# Single-Image Super-Resolution via Cascaded Non-Local Mean Network and Dual-Path Multi-Branch Fusion

**DOI:** 10.3390/s25134044

**Published:** 2025-06-28

**Authors:** Yu Xu, Yi Wang

**Affiliations:** School of Computer and Control Engineering, Yantai University, Yantai 264005, China; 1546097417@s.ytu.edu.cn

**Keywords:** image super-resolution, self-attention, CNN, non-local means, multi-branch fusion

## Abstract

Image super-resolution (SR) aims to reconstruct high-resolution (HR) images from low-resolution (LR) inputs. It plays a crucial role in applications such as medical imaging, surveillance, and remote sensing. However, due to the ill-posed nature of the task and the inherent limitations of imaging sensors, obtaining accurate HR images remains challenging. While numerous methods have been proposed, the traditional approaches suffer from oversmoothing and limited generalization; CNN-based models lack the ability to capture long-range dependencies; and Transformer-based solutions, although effective in modeling global context, are computationally intensive and prone to texture loss. To address these issues, we propose a hybrid CNN–Transformer architecture that cascades a pixel-wise self-attention non-local means module (PSNLM) and an adaptive dual-path multi-scale fusion block (ADMFB). The PSNLM is inspired by the non-local means (NLM) algorithm. We use weighted patches to estimate the similarity between pixels centered at each patch while limiting the search region and constructing a communication mechanism across ranges. The ADMFB enhances texture reconstruction by adaptively aggregating multi-scale features through dual attention paths. The experimental results demonstrate that our method achieves superior performance on multiple benchmarks. For instance, in challenging ×4 super-resolution, our method outperforms the second-best method by 0.0201 regarding the Structural Similarity Index (SSIM) on the BSD100 dataset. On the texture-rich Urban100 dataset, our method achieves a 26.56 dB Peak Signal-to-Noise Ratio (PSNR) and 0.8133 SSIM.

## 1. Introduction

Image resolution refers to the amount of spatial detail captured in an image, typically quantified by the number of pixels per unit area. Higher resolution enables finer visual details to be distinguished, which is essential for accurate interpretation and analysis in various computer vision and image processing tasks. In many real-world applications—such as medical diagnostics, remote sensing, security surveillance, and autonomous driving—HR images are important. They help to identify subtle patterns, detect small objects, and improve the reliability of downstream algorithms [1,2,3,4]. For instance, in medical imaging, a higher resolution can reveal microstructures that are vital for early disease detection; in autonomous driving, it can enhance object recognition accuracy for better decision-making.

However, directly capturing HR images through physical sensors remains challenging due to multiple factors. These include limitations in sensor size and pixel density, optical aberrations and diffraction, motion-induced blur, bandwidth constraints, storage requirements, and cost [5,6,7]. For example, miniaturized imaging devices often rely on compact sensors with small pixel sizes, which restrict light sensitivity and increase noise. Optical imperfections further degrade image sharpness, while high-speed scenes may introduce motion blur. Additionally, transmitting or storing HR images is often infeasible in resource-constrained environments. Due to these limitations, many imaging systems produce low-LR outputs, limiting their effectiveness in practical applications.

To address this problem, SR techniques have been developed as computational alternatives to hardware-based solutions. SR aims to reconstruct HR images from one or more LR observations, enhancing visual fidelity and structural details without changing the image acquisition hardware. Compared to sensor upgrades, SR methods are more cost-effective and flexible, and have become an essential preprocessing step for tasks like object detection [8], segmentation [9], and scene understanding [5].

The early SR methods addressed this ill-posedness by introducing prior knowledge of natural image statistics into the reconstruction process [10]. These approaches typically added regularization terms to the objective function to favor plausible solutions [11,12]. The common regularization techniques include L1 regularization (encouraging sparsity in the image gradient), L0 regularization (promoting even sparser gradients), mixed hybrid L1–L0 regularization, and sparse representation (assuming that image patches can be represented as a sparse linear combination of atoms from a learned dictionary) [11,13]. While these methods offered some degree of improvement in image resolution, they often failed to capture the complex nonlinear patterns present in natural scenes. This limitation frequently resulted in artifacts such as blurring, ringing, and the loss of fine textures.

The introduction of deep learning, particularly convolutional neural networks (CNNs), advanced the performance of SISR. CNNs can learn hierarchical features and model complex nonlinear mappings. They have achieved much better results than traditional approaches [14,15,16,17,18,19,20,21,22,23], largely because they can automatically extract informative patterns from large datasets. CNNs also exploit spatial correlations within small image regions, making them effective for local feature extraction [14,16,18,19]. Due to their relatively small number of parameters compared to fully connected networks, CNNs are also more computationally efficient. However, a limitation of standard CNNs is their limited receptive field. This limits their ability to capture long-range dependencies, which are important for understanding global context, identifying repeated textures, and reconstructing structured objects.

To overcome this, researchers have turned to Transformers. Their multi-head self-attention mechanism allows each pixel to interact with all the other pixels in the image, regardless of spatial distance [24,25,26,27,28]. This enables modeling of long-range dependencies and global structure. Despite their strengths, Transformers face scalability issues. The self-attention mechanism has quadratic complexity with respect to image size, making standard Transformers computationally expensive. This high cost requires large training datasets and powerful Graphics Processing Units (GPUs), which can limit their usability in practical scenarios, particularly on resource-constrained devices. Although many lightweight Transformer variants have been proposed to mitigate these computational costs [29,30,31,32], they often trade performance for efficiency. As a result, they may struggle to reconstruct fine details and textures.

Given the respective strengths and limitations of CNNs and Transformers, hybrid CNN–Transformer architectures have emerged as a promising research direction for SISR. However, the existing hybrid methods still struggle to effectively integrate long-range dependencies with fine-grained local details. To address this gap, we propose a novel hybrid architecture called PSNLMN. The key innovations of our approach are as follows:

1. We introduce a novel PSNLM module, inspired by the traditional NLM algorithm, which effectively captures fine-grained long-range dependencies by combining the strengths of NLM and self-attention mechanisms;
2. We design an ADMFB that enhances both scale diversity and feature discriminability by simultaneously extracting and fusing hierarchical features through parallel attention pathways;
3. We integrate a PSNLM and ADMFB into a CNN framework, achieving competitive performance on standard benchmarks, demonstrating the effectiveness of our hybrid design.

To the best of our knowledge, this is the first attempt to combine the principles of NLM with self-attention mechanisms for the task of image super-resolution. To clearly present our approach and findings, the remainder of this paper is organized as follows: Section 1 introduces the background, motivation, and contributions of the study. Section 2 provides a detailed review of the related work, including the existing SISR methods and their limitations. Section 3 elaborates on our proposed PSNLMN, including the network framework and the design details and principles of the NLMB, PSNLM, and ADMFB. Section 4 describes the experimental settings, ablation studies, and performance evaluations. Section 5 concludes the paper by summarizing the proposed method and contributions.

## 2. Related Work

SR has been an active research area for decades, with a wide range of approaches proposed to address this challenging problem. These methods are typically divided into two categories: traditional approaches and deep learning-based methods.

### 2.1. Traditional Methods

Traditional SR techniques can be broadly categorized into interpolation-based, reconstruction-based, and example-based methods. Interpolation-based methods, such as bicubic interpolation [33] and Lanczos resampling [34], are computationally efficient. However, they assume local continuity in the image signal, which often leads to oversmoothing. Consequently, these methods tend to blur edges and lose high-frequency details [35], limiting their ability to preserve fine textures.

Reconstruction-based methods were developed to address the shortcomings of interpolation. They formulate SISR as an inverse problem and incorporate image priors to regularize the solution. These priors are introduced through objective functions that balance data fidelity with regularization terms, such as Tikhonov regularization, Total Variation (TV), or sparsity-promoting norms like $l_{1}$ and $l_{2}$ minimization [12,36,37]. Although these methods often produce sharper results, they are sensitive to the choice in regularization parameters, which can be difficult to tune and may not generalize well to different image types [38]. Moreover, the use of hand-crafted priors limits their ability to capture the complex and nonlinear structures found in natural images, often resulting in artifacts and limited texture reconstruction.

Example-based SR represents a learning-based alternative. These methods attempt to learn mappings from low-resolution to high-resolution patches using either external dictionaries (e.g., sparse coding [13]) or internal image similarities (e.g., anchored neighborhood regression [39]). Although capable of producing visually pleasing details, example-based methods face two main challenges: (1) high computational cost during patch matching and (2) strong dependence on the quality and relevance of the training data or the presence of repetitive patterns within the input image.

### 2.2. Deep Learning-Based Super-Resolution Methods

The development of deep learning has brought significant progress to the field of SR. The methods based on deep learning can be broadly divided into three categories: CNN-based, Transformer-based, and hybrid CNN–Transformer approaches.

#### 2.2.1. CNN-Based Methods

Dong et al. [14] proposed the Super-Resolution Convolutional Neural Network (SRCNN), which was the first to apply CNNs to SISR. The SRCNN uses a shallow three-layer network to learn a nonlinear mapping from LR to HR image patches. Although it outperforms traditional methods, the SRCNN is limited by its small receptive field, which hinders its ability to capture long-range dependencies within images.

To improve performance, later works introduced deeper CNN architectures. Kim et al. [40] presented the Very Deep Super-Resolution (VDSR) network, which uses a 20-layer structure with residual learning to enhance reconstruction accuracy and ease training. Tai et al. [41] proposed the Deep Recursive Residual Network (DRRN), which employs recursive blocks to deepen the network without significantly increasing the number of parameters.

Deeper networks generally improve reconstruction quality, but they can also be harder to train. To address this and to enhance feature representation, attention mechanisms have been incorporated. Zhang et al. [19] introduced the Residual Channel Attention Network (RCAN), which applies channel attention to adaptively reweight feature maps. This helps the network to focus on more informative channels.

CNN-based methods are effective in extracting local features. However, due to the localized nature of convolution, they face challenges in modeling long-range dependencies, which are important for capturing global image context.

#### 2.2.2. Transformer-Based Methods

Motivated by the success of Transformers in natural language processing, researchers have adapted them for image processing tasks, including SR. Chen et al. [42] proposed the Image Processing Transformer (IPT), a large-scale pre-trained model that applies the Transformer architecture to various low-level vision problems. IPT demonstrated strong performance in SR tasks. However, the computational cost of the standard Transformer scales quadratically with image resolution, making it both memory- and computation-intensive.

To mitigate this, Liang et al. [26] introduced SwinIR, which is built on the Swin Transformer [25]. It employs shifted windows to reduce computational complexity while retaining the ability to model long-range dependencies. In addition, several lightweight Transformer architectures have been proposed to further reduce resource demands. Despite improvements in efficiency, these models often face trade-offs in reconstruction quality, especially in recovering fine details and textures.

#### 2.2.3. Hybrid CNN–Transformer Methods

To leverage the strengths of both CNNs and Transformers, recent studies have explored hybrid architectures that combine local feature extraction with global context modeling. These approaches aim to integrate the localized processing capabilities of CNNs with the broader receptive fields of Transformers. This combination enhances reconstruction quality by addressing both local and global information.

For instance, the ESRT [31] embedded Transformer modules within a CNN framework to balance computational efficiency with contextual awareness. LBNet [32] introduced bimodal blocks that capture both local patterns and non-local dependencies. Methods such as SwinIR [26] and its variants [28] have shown good performance on several benchmark datasets, highlighting the potential of hybrid designs.

Despite these advancements, many hybrid models still struggle with the reconstruction of high-frequency details and complex textures. Although Transformers help in capturing global structure, the current fusion mechanisms or attention designs may not fully preserve local fine-grained features. As a result, reconstructed images can lack detail clarity, particularly in areas containing dense textures or repetitive patterns.

Therefore, there is a continued need for hybrid architectures that better integrate global and local information. Such designs should aim to improve the fidelity of texture and detail reconstruction without compromising efficiency.

## 3. Methods

### 3.1. Network Structure

**Figure 1:**
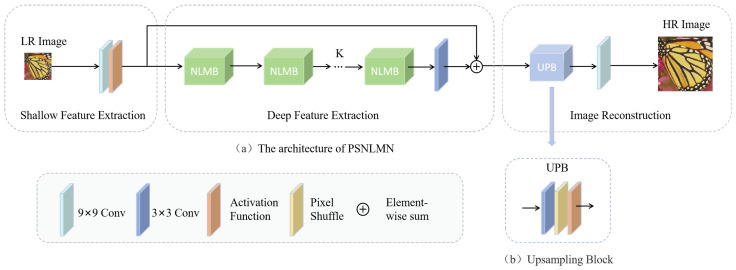
The overall overview of the proposed PSNLMN. (**a**) The architecture of PSNLMN; (**b**) upsampling block.

The proposed network framework is illustrated in Figure 1a. It consists of two main stages: feature extraction and image reconstruction. The feature extraction stage is further divided into shallow feature extraction and deep feature extraction.

Shallow Feature Extraction: Initially, the input image $I_{LR}\in R^{H\times W\times C_{in}}$ undergoes shallow feature extraction using a single convolutional layer with a kernel size of 9×9. This layer aims to capture low-level spatial information and enhance the initial representation of the input image. The process can be represented as

(1) $$F_{s}=\sigma_{prleu}\left(C_{9\times 9}\left(I_{LR}\right)\right)$$

where $F_{s}\in R^{H\times W\times C}$ denotes the shallow features, $\sigma_{prleu}$ is the PReLU activation function, and $C_{9\times 9}\left(\cdot \right)$ represents a convolutional operation with a 9×9 kernel. The 9×9 kernel provides a large receptive field for capturing contextual information at an early stage.

Deep Feature Extraction: The shallow features $F_{s}$ serve as input to the deep feature extraction module. This module adopts a cascaded structure of *k* non-local multi-branch modules (NLMBs). These modules are designed to effectively capture both global and local features at multiple scales. The deep feature extraction can be formulated as

(2) $$F_{d}^{\prime }=C_{3\times 3}\left(NLMB_{k}\left(NLMB_{k-1}\left(\ldots NLMB_{1}\left(F_{s}\right)\ldots \right)\right)\right)$$

where $NLMB_{i}$ represents the i-th NLMB block in the cascade and $C_{3\times 3}\left(\cdot \right)$ represents the 3×3 convolutional operation. The output feature $F_{d}^{\prime }$ is then combined with the input shallow features through a residual connection:

(3) $$F_{d}=F_{s}+F_{d}^{\prime }$$

where $F_{d}\in R^{H\times W\times C}$ is the final output of the deep feature extraction stage. This residual connection facilitates gradient flow during training and allows the network to learn residual information, further enhancing the feature representation.

Image Reconstruction: The image reconstruction stage focuses on upsampling deep feature maps $F_{d}$ to the desired output resolution. This is achieved through the use of upsampling block (UPB), as detailed in Figure 1b. The upsampling operation can be represented as

(4) $$F_{up}=UPB\left(F_{d}\right)=\sigma_{prleu}\left(Pixel\;Shuffle\left(C_{3\times 3}\left(F_{d}\right)\right)\right)$$

where PixelShuffle operation efficiently upsamples feature maps by rearranging channels into spatial resolution [43], $F_{up}\in R^{(rH)\times (rW)\times C}$ is the output of the UPB, and *r* is the upscaling factor for super-resolution.

Following the upsampling process, a final convolutional layer with a 9×9 kernel is applied to refine the reconstructed image. This final convolutional layer mirrors the initial shallow feature extraction layer, allowing for effective feature fusion and adaptation of the reconstructed features to the desired output characteristics:

(5) $$I_{HR}=C_{9\times 9}\left(F_{up}\right)$$

where $I_{HR}\in R^{\left(rH\right)\times \left(rW\right)\times C_{in}}$ is the final reconstructed HR image.

### 3.2. The Non-Local Multi-Branch Module (NLMB)

**Figure 2:**
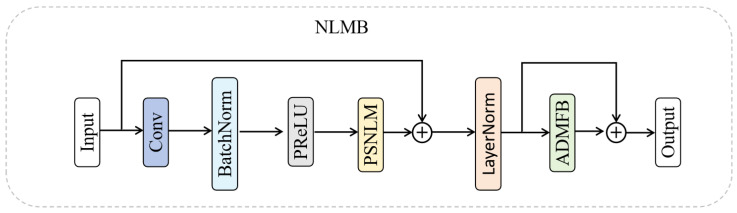
The non-local multi-branch module (NLMB).

The NLMB, depicted in Figure 2, is a core component designed to extract and fuse both global and local features. The module consists of three primary parts: initial feature extraction, PSNLM, and ADMFB.

In initial feature extraction, input features $X\in R^{H\times W\times C}$ are processed through a 3×3 convolutional layer and followed by batch normalization (BN) and a PReLU activation function to enhance the representation, as shown in Equation (6). This initial processing provides a feature foundation for subsequent stages. The resulting feature map is then fed into a PSNLM, which captures long-range dependencies and global contextual information; more details of PSNLM will be given in Section 3.3. The output $F_{PSNLM}$ of the PSNLM block is combined with the initial feature map using a residual connection and is followed by layer normalization (LN) to stabilize the training, as described by Equation (7).

(6) $$F_{in}=\sigma_{prleu}\left(BN\left(C_{3\times 3}\left(X\right)\right)\right)$$

(7) $$F_{LN}=LN\left(X+F_{PSNLM}\right)$$

(8) $$\;F_{NLMB}=F_{ADMFB}+F_{LN}$$

In the final stage, the normalized features are fed into the ADMFB block (further details will be provided in Section 3.4), which extracts and fuses multi-scale features across branches. A final skip connection refines the module output, as shown in Equation (8), where $F_{NLMB}\in R^{H\times W\times C}$ represents the final output feature map of the NLMB module. This skip connection allows the module to learn residual features and enhance the output representation.

### 3.3. Pixel-Wise Self-Attention-Based Non-Local Mean Module (PSNLM)

**Figure 3:**
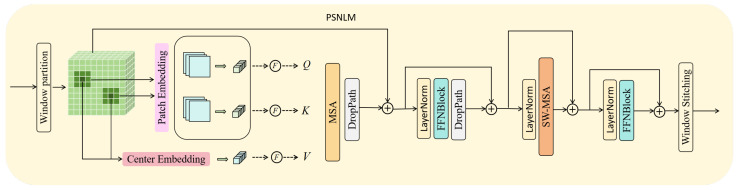
Pixel-wise self-attention-based non-local mean module (PSNLM).

The architecture of PSNLM is illustrated in Figure 3. By integrating NLM with pixel-wise attention, it enables efficient modeling of long-range dependencies. Traditional NLM algorithms perform a pixel-wise search across the entire image to compute similarities, resulting in substantial computational redundancy and high complexity. This is particularly problematic for high-resolution images. To mitigate this, we adopt a window-based partitioning strategy that limits the search scope, reducing computational burden and enabling efficient high-resolution processing. Furthermore, the use of fixed-size windows facilitates parallel computation, improving the overall efficiency of the algorithm.

**Figure 4:**
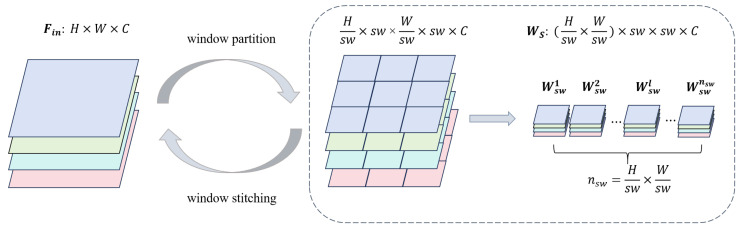
Window partition and window stitching. Different colors represent different channels.

Specifically, as illustrated in Figure 4, the input feature map $F_{in}$ is initially divided into $n_{sw}$ non-overlapping equally sized sub-feature maps $W_{s}=\left\{W_{sw}|W_{sw}\in R^{sw\times sw\times C}\right\}$ via a window partitioning operation. Each sub-feature map, of size sw×sw×C, is treated as an independent search window, where sw represents the search window size.

Within each search window, we adapt the core concept of NLM, where similarities are assessed based on the similarity between local neighborhoods centered around individual pixels. We draw inspiration from the Transformer architecture by incorporating learned embeddings for these neighborhoods. Specifically, we first define a local neighborhood window $W_{nw}\in R^{nw\times nw\times C}$. Depth-wise convolutional and point-wise convolutional operations are then employed to extract features from these neighborhoods. The embedding for each neighborhood patch is obtained as follows:

(9) $$f_{w_{j}}=F\left(\sigma_{l}\left(C_{point}\left(C_{depth}\left(W_{nw}^{j}\right)\right)\right)\right)$$

where *F* is the flatten operation, $\sigma_{l}$ denotes the LeakyReLU activation function, $C_{point}$ represents a point-wise 1×1 convolutional operation, $C_{depth}$ represents a depth nw×nw convolutional operation, and $f_{w_{j}}\in R^{1\times d}$ is the embedded features of the j-th neighborhood patch, where *d* represents the number of kernels in the convolution operation, and the maximum value of *j* is sw×sw, which reflects the number of domain blocks within the search window.

For the center point $X_{j}\in R^{1\times 1\times C}$ within each neighborhood patch, we extract a feature vector using a point-wise convolutional operation:

(10) $$c_{j}=F\left(\sigma_{l}\left(C_{point}\left(x_{j}\right)\right)\right)$$

where $c_{j}\in R^{1\times C}$ is the embedding vector representing the center point.

Given a query point $X_{q}$ within a search window $W_{sw}^{l}\in W_{s}$, the similarity computation proceeds as follows: the embedding vector $f_{w_{q}}$ for the neighborhood patch and the embedding vector $c_{q}$ for the center point $X_{q}$ are computed using Equations (9) and (10), respectively. Notably, zero-padding with a width of $\frac{nw}{2}$ is applied around the search window to ensure that query points positioned at or near the window boundaries maintain a complete nw×nw neighborhood, thereby guaranteeing robust algorithm execution and processing. Similarly, we extract the features $W_{k}=\left\{f_{w_{j}}\right\}$ for all patches within $W_{sw}^{l}$ using Equation (9), with corresponding center points $W_{v}=\left\{c_{j}\right\}$. Next, these embedded features are distributed across *n* attention heads, as follows:

(11) $$\begin{array}{cc} \; & f_{w_{q}}=\left[f_{w_{q}}^{1},f_{w_{q}}^{2},\ldots ,f_{w_{q}}^{n}\right],\;W_{k}=\left[W_{k}^{1},W_{k}^{2},\ldots ,W_{k}^{n}\right],\;W_{v}=\left[W_{v}^{1},W_{v}^{2},\ldots ,W_{v}^{n}\right] \end{array}$$

where $f_{w_{q}}^{i}\in R^{1\times \frac{d}{n}}$, $W_{k}^{i},W_{v}^{i}\in R^{\left(sw\cdot sw\right)\times \frac{d}{n}}$, and the superscript i denotes the i-th attention head. The similarity between all points $C=\{X_{j}|j=1,2,\ldots ,sw\cdot sw\}$ in $W_{sw}^{i}$ and the query point $X_{q}$ is then computed using a dot product operation:

(12) $$s^{i}\left(x_{q},C\right)=s^{i}\left(f_{w_{q}},W_{k}\right)=Softmax\left(\frac{f_{w_{q}}^{i}\cdot {\left(W_{k}^{i}\right)}^{T}}{\sqrt{d/n}}\right)$$

where $\sqrt{d/n}$ is the scaling factor, Softmax is the normalized function, and $s^{i}\in R^{1\times (sw\cdot sw)}$ represents the similarity score between the query point and all pixels. Then, we weight $W_{v}$ by similarity as follows:

(13) $$o^{i}=s^{i}\left(x_{q},C\right)\cdot W_{v}=\sum_{j=1}^{sw\times sw}s\left(x_{q},x_{j}\right)\cdot c_{j}$$

where $O^{i}$ is the weighted output of the i-th attention head. Subsequently, we fuse the multiple heads using a set of weights to obtain the final attention output:

(14) $$c_{q}^{out}=Concat\left(o^{1},o^{2},\ldots ,o^{i},\ldots ,o^{n}\right)W_{o}$$

where $c_{q}^{out}\in R^{1\times C}$, Concat denotes the concatenation function employed to concatenate the outputs from multiple attention heads along a specified dimension, and $W_{o}$ is a linear layer function used to aggregate the outputs of multiple heads and subsequently map them back to the original channel dimension. Finally, a spatial restoration transformation $F^{-1}$ is utilized to reconstruct the output $x_{q}^{out}\in R^{1\times 1\times C}$ to its original spatial dimensions:

(15) $$x_{q}^{out}=F^{-1}\left(c_{q}^{out}\right)$$

By iterating this process for all query points within the search window, we obtain the output feature map $W_{s}^{out}$ for each window. These feature maps are then normalized using layer normalization. Next, they are processed by a feedforward network consisting of convolutional layers and a DropPath layer, producing an intermediate result. The normalization stabilizes the feature distribution, while the convolutional layers capture local spatial patterns. The DropPath mechanism is incorporated to enhance regularization and prevent overfitting.

To enable information exchange between different windows, we adopt the Shifted Windows Multi-Head Self-Attention (SW-MSA) mechanism [25]. The output of SW-MSA is further processed by another sequence of layer normalization and a convolutional feedforward network, generating the final feature maps. This repeated structure maintains consistency across the network. Finally, the output feature maps of all search windows are stitched, as shown in Figure 4, forming a global feature map with the same spatial dimensions as the input.

Note that we use the dot product in the attention mechanism to measure similarity instead of the nonlinear Gaussian kernel (used in NLM algorithm). This choice is based on the observation that the features undergo multiple nonlinear transformations throughout the network, allowing the dot product to implicitly model complex relationships.

### 3.4. Adaptive Dual-Path Multi-Branch Fusion Block (ADMFB)

**Figure 5:**
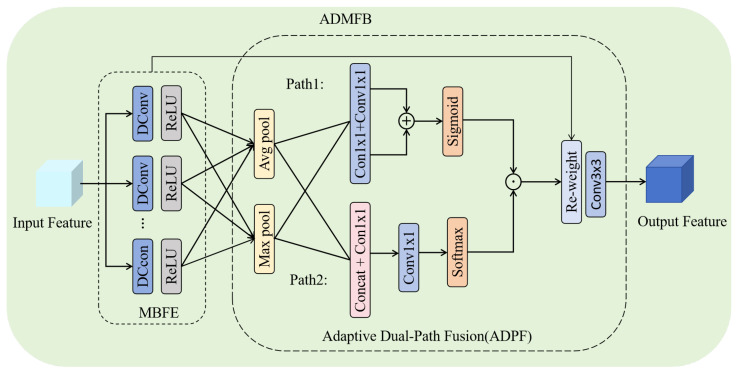
Adaptive dual-path multi-branch fusion block (ADMFB).

To improve multi-scale feature representation in SR, we propose ADMFB, a novel architecture that aggregates features at different scales. As depicted in Figure 5, the ADMBFM comprises two components: a multi-branch feature extraction (MBFE) block and an adaptive dual-path fusion (ADPF) block. The MBFE uses multiple branches with dilated convolutions at different rates. This design captures contextual information across scales, addressing scale variation in image processing. The subsequent ADPF module is responsible for adaptively fusing the features extracted by the MBFE unit. This fusion is achieved through a dual-path approach to leverage complementary information. The specific details are as follows:

The MBFE begins with distributing the input feature $Y\in R^{H\times W\times C}$ to *N* parallel branches. Each branch extracts scale-specific features using dilated convolutions $Y_{i}^{\prime }=\phi \left(Y;d_{i}\right)$, where $Y_{i}^{\prime }\in R^{H\times W\times C}$, $d_{i}$ represents the dilation rate of the i-th branch and ϕ(·) describes dilated convolution operation. The multi-branch structure generates feature maps enriched with diverse scales.

In ADPF, the feature maps captured from MFBE are sent to average pooling and maximum pooling layers; the results are addressed as $P_{avg}$ and $P_{max}\in R^{1\times 1\times C}$, respectively. $P_{avg}$ and $P_{max}$ are then passed through two parallel attention generation paths to compute adaptive weights:

Path 1: The global features are processed by two shared 1 × 1 convolution layers, followed by the element-wise addition and an activation function, generating channel attention weights:

(16) $$W_{i}^{\left(1\right)}=\sigma \left(f_{1b}\left(f_{1a}\left(P_{i}^{avg}\right)\right)+f_{1b}\left(f_{1a}\left(P_{i}^{max}\right)\right)\right)$$

where $f_{1a}\left(\cdot \right)$ and $f_{1b}\left(\cdot \right)$ represent two distinct sequential 1 × 1 convolution operations. The former reduces the channel dimensionality to $C/r_{1}$, while the latter expands it back to C, $W_{i}^{\left(1\right)}\in R^{1\times 1\times C}$, and σ(·) denotes the sigmoid activation function. This path employs channel attention mechanisms to selectively weight each branch of the MBFE, thereby emphasizing the informative features and suppressing irrelevant noise.

Path 2: The pooled features are first concatenated and then compressed through a 1 × 1 convolution. Subsequently, an additional convolution is applied, followed by normalization using the softmax function to generate another set of weights.

(17) $$W_{i}^{\left(2\right)}=\frac{f_{2b}\left(f_{2a}\left(Concat\left(P_{i}^{avg},P_{i}^{max}\right)\right)\right)}{\sum_{j=1}^{N}f_{2b}\left(f_{2a}\left(Concat\left(P_{j}^{avg},P_{j}^{max}\right)\right)\right)}$$

where $W_{i}^{\left(2\right)}\in R^{1\times 1\times C}$ is the result of normalizing with respect to the other weights, *N* denotes the number of branches, $f_{2a}\left(\cdot \right)$ and $f_{2b}\left(\cdot \right)$ represent other group 1 × 1 convolution operations, the first 1 × 1 convolution reduces the channel dimension from 2C to $\frac{2C}{r_{2}}$, and the second 1 × 1 convolution reduces it to C. Path 2 is designed to calculate fusion weights that govern the contribution of each branch during feature aggregation.

Ultimately, the weights from the two paths are multiplied ($W_{i}=W_{i}^{\left(1\right)}⊙W_{i}^{\left(2\right)}$) to compute the final attention weight for each branch, producing the weighted feature $Y_{i}^{\prime \prime }=Y_{i}^{\prime }⊙W_{i}$. A final 3×3 convolution is applied to further refine and integrate the details, and to generate the output feature $Y_{output}=C_{3\times 3}\left(\sum_{i=1}^{N}Y_{i}^{\prime \prime }\right)$.

In summary, ADMFB allows the block to dynamically prioritize branches based on their relevance to the specific input, mitigating the limitations of static fusion strategies. By combining these two paths, the ADMFB effectively achieves a more nuanced and context-aware fusion process, leading to enhanced feature representation and improved performance in reconstruct tasks. This dual-path design combines channel-wise selection and adaptive branch weighting, enabling the network to model dependencies across scales.

## 4. Experiments

### 4.1. Experimental Settings

We trained the PSNLMN on an NVIDIA RTX 3090 GPU using 120K randomly sampled images from the CoCo2014 dataset [44]. To evaluate model performance, we conducted experiments on four widely used benchmark datasets: Set5 [45], Set14 [46], BSD100 [47], and Urban100 [48]. The super-resolution performance was assessed by calculating the PSNR and SSIM on the luminance (Y) channel. The detailed training configuration and parameter settings are described as follows:

During the training, LR images were generated by downsampling HR images using bicubic interpolation, followed by data augmentation through random horizontal flips and orthogonal rotations (90° and 270°). LR patches were extracted at 48 × 48 pixel resolution. Network parameters were initialized via Kaiming initialization with an initial learning rate of 0.0002. Optimization employed the AdamW optimizer with momentum coefficients $\beta_{1}=0.9$ and $\beta_{2}=0.999$, minimizing the $L_{2}$ loss function. Models were trained for 300 epochs with a batch size of 64, maintaining a constant learning rate for the initial 150 epochs before applying linear decay. After each epoch, model performance was validated and weights archived. The optimal model was selected post-training through comprehensive evaluation of loss convergence trajectories and validation metrics.

Testing comprised (1) loading preprocessed LR test data into the best-trained model; (2) generating super-resolved outputs; and (3) computing quantitative metrics against ground-truth HR references. A batch size of 1 was implemented during testing to eliminate spatial variance in metric computation.

The proposed network employs a base configuration with 64 channels (C = 64), incorporating 4 attention heads, an 8 × 8 search window, and a neighborhood window size of 3. The ADFB block utilizes reduction factors *r* = 16. For ×4 super-resolution, the architecture stacks 10 NLMBs, where the ADMFB features three parallel branches with dilation rates of 1, 2, and 3, respectively. When the scaling factor is reduced to ×2 or ×3, we adjust the number of NLMBs to 12 and expand the ADMFB to four parallel branches with dilation rates of 1, 2, 3, and 4, thereby enhancing multi-scale feature extraction capabilities.

### 4.2. Ablation Studies

In this section, we conduct ablation studies on the two core components of our proposed PSNLMN—PSNLM and ADMFB—to validate their effectiveness. To ensure fairness, all experiments use the same parameters (learning rate, optimizer, batch size, network architecture, etc.) and data preprocessing techniques as used in the original model training. For efficiency, the models are trained for only 100 epochs with a magnification factor of 4 and evaluated on the same test dataset. Although the reported metrics may be lower than those of the fully trained models in the quantitative result analysis, this accelerated evaluation enables rapid assessment of different configurations and reveals the contributions of individual components. The observed trends and relative performance differences are consistent, meaningful, and indicative of each component’s value.

#### 4.2.1. Ablation Study of Module PSNLM

**Figure 6:**
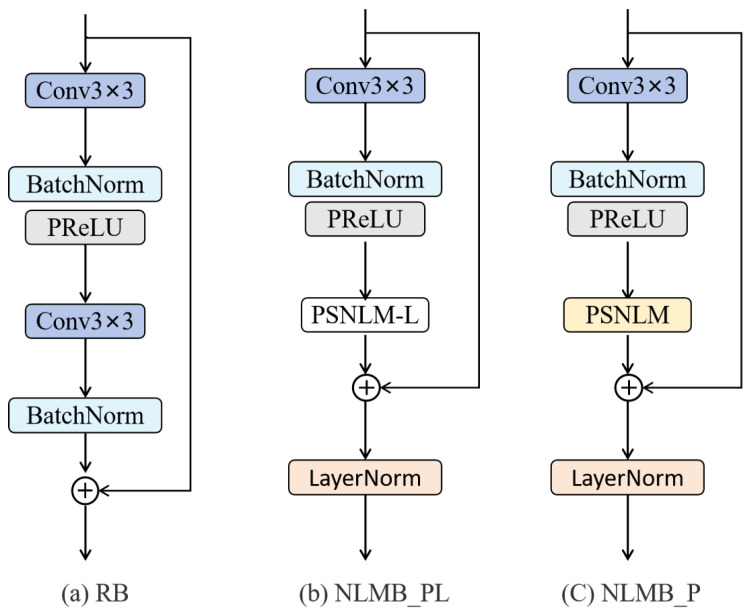
The schematic diagram of the structures of RB, NLMB_PL, and NLMB_P.

To establish a baseline, we replaced the NLMB in the deep feature extraction stage with a classical residual block (RB) [16] and named it Base_Model. The RB consists of two convolutional layers and a residual connection, as shown in Figure 6a.

To verify the effectiveness of the proposed PSNLM module, we replace the second convolutional layer in the RB module with our PSNLM and add a layer normalization, as shown in Figure 6c. This modified block is named NLMB_P and is used to construct the model Model_PSNLM. In addition, to investigate the impact of the window interaction mechanism, we replace the PSNLM in NLMB_P with a variant that does not include window interaction, referred to as PSNLM_L, as shown in Figure 6b. We name this block NLMB_PL, which is used to construct the model Model_PSNLM_L.

According to Table 1, Model_PSNLM_L, which includes an incomplete version of PSNLM, achieves modest improvements in both PSNR and SSIM over the baseline model across all four benchmark datasets. For example, on the Urban100 dataset, Model_PSNLM_L improves the PSNR from 25.670 dB to 26.098 dB (+0.428 dB) and SSIM from 0.7870 to 0.7994 (+0.0124). These results suggest that the core design of PSNLM, even in its simplified form, is more effective than traditional residual blocks for capturing and reconstructing image features, particularly in images with complex structures. Furthermore, comparing Model_PSNLM_L with Model_PSNLM, which includes the complete PSNLM module, we observe further performance improvements. For example, on the BSD100 dataset, PSNR increases from 27.561 dB to 27.613 dB (+0.052 dB).

Overall, these results demonstrate that the model incorporating the complete PSNLM module offers comprehensive performance advantages over the baseline.

#### 4.2.2. Ablation Study of Module ADMFB

To effectively capture multi-scale features, an effective strategy involves the parallel employment of dilated convolution layers with varying dilation rates. This approach has been successfully implemented in the Wide-Focus [27], which integrates contextual information across different ranges by combining standard convolution with multi-branch dilated convolutions, followed by additive fusion. However, this architecture has two limitations: (1) insufficient channel-wise selectivity within individual branches, and (2) the assumption of fixed contribution weights across different scales due to the simplistic element-wise summation used during fusion. The proposed ADMFB addresses these shortcomings through two key innovations: Firstly, Path 1 enables adaptive enhancement of informative channels and suppression of redundancy within each branch through channel-wise attention mechanisms, thereby optimizing intra-scale feature representation before fusion. Secondly, Path 2 replaces conventional additive fusion with content-aware adaptive weighting, allowing dynamic adjustment of cross-scale contribution weights based on input characteristics.

**Figure 7:**
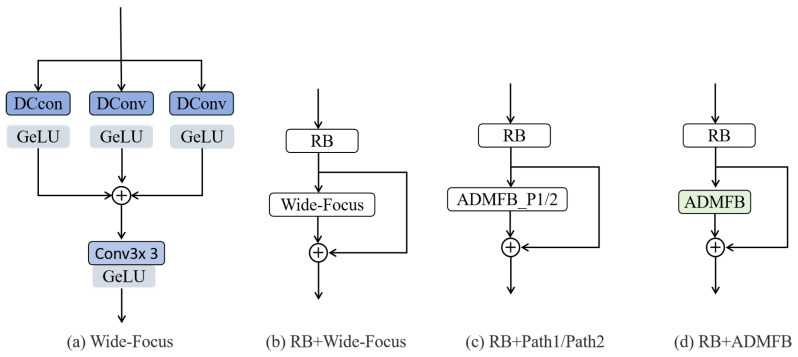
The schematic diagram of the structures of Wide-Focus and the basic blocks used in the experiment.

To quantitatively assess the contribution of each path, we conducted ablation studies. We constructed variants by incorporating simplified modules containing only the Path 1 components or only the Path 2 components into the baseline architecture, designated Base_Model_P1 and Base_Model_P2, respectively (illustrated by components in Figure 7c). Furthermore, to validate the overall efficacy of our proposed ADMFB against the original Wide-Focus, we established comparative baseline models: Base_Model_WF, which incorporates the original Wide-Focus block (Figure 7a), and Base_ADMFB, incorporating our complete ADMFB block (Figure 7b). The corresponding experimental results are presented in Table 2.

The ablation studies conducted across four benchmark datasets quantitatively validate the effectiveness of our adaptive dual-path fusion strategy. Compared to the baseline Wide-Focus model (Base_Model_WF), the incorporation of Path 1’s adaptive channel attention mechanism (Base_Model_P1) yields consistent, although moderate, improvements across all datasets. These enhancements are particularly pronounced in texture-rich Urban100. This demonstrates its capability in enhancing intra-scale feature discriminability through channel-wise feature refinement. Path 2’s dynamic fusion mechanism (Base_Model_P2) exhibits more substantial performance gains, especially in complex urban scenarios (Urban100) and natural imagery (BSD100), confirming the critical role of context-aware cross-scale integration. The complete ADMFB (Base_ADMFB) achieves optimal performance through synergistic combination of both pathways, showing progressive metric improvements that positively correlate with dataset complexity. Notably, the most significant performance gap between ADMFB and Wide-Focus emerges in Urban100, where structural complexity necessitates precise channel selection and adaptive multi-scale fusion. The consistent performance gains across datasets—from simple (Set5) to complex (Urban100)—highlight the complementary strengths of our dual-path design. Path 1 enhances intra-scale representation via adaptive channel recalibration, while Path 2 prioritizes cross-scale context using learnable weight allocation. This hierarchical design enables selective integration of the most relevant multi-scale features, enhancing image reconstruction quality. The experimental evidence suggests that the dual-path collaboration effectively addresses the limitations of fixed fusion paradigms by jointly improving intra-scale discrimination and cross-scale context modeling.

#### 4.2.3. Ablation Study on the Combined Modules

The previous sections have confirmed the individual contributions of PSNLM and ADMFB. To further explore the overall gain achieved by their combination, we conducted joint ablation experiments, as summarized in Table 3, to assess the combined effect of both modules on super-resolution performance. Specifically, PSNLMN is constructed by replacing the RB module in Model_ADMFB with NLMB_P. In addition, we investigate how varying the number of branches (num_branch) in the MBFB module of ADMFB affects the performance of the combined model. The corresponding results are also summarized in Table 3.

Comparison between Model_ADMFB, Model_PSNLM, and the integrated model PSNLM_3 (three-branch configuration) shows that the integrated model PSNLMN_3, incorporating both ADMFB and PSNLM modules, achieved the highest PSNR/SSIM metrics across all four benchmark datasets. Specifically, on the Set14 dataset, the PSNR performance of PSNLMN_3 exhibited improvements of 0.322 dB over Model_PSNLM and 0.359 dB over Model_ADMFB. This finding underscores that the integration of both modules demonstrates effective complementarity in capturing multi-scale features and modeling long-range dependencies.

Furthermore, systematic comparisons of reconstruction performance under varying branch numbers in MBFB reveal that the 3-branch configuration (PSNLMN_3) consistently achieves optimal PSNR/SSIM metrics across all test datasets. Increasing the number of branches from 2 to 3 led to notable performance gains, particularly on Set14 and Urban100. The 3-branch configuration also showed more stable or slightly better performance than the 4-branch configuration. This pattern suggests that the 3-branch architecture optimally balances feature extraction efficiency and fusion effectiveness in the PSNLMN framework. Too few branches can limit multi-scale feature representation, while too many may introduce redundancy or training instability without meaningful performance gains, sometimes even causing slight degradation.

Collectively, the experiments validate the efficacy of combining ADMFB with PSNLM, while quantitatively establishing the 3-branch MBFB configuration as the optimal architectural choice under current magnification factors.

### 4.3. Performance Evaluation

To assess the effectiveness of the proposed PSNLMN, we conducted comparisons with several state-of-the-art SR methods, including MemNet [15], SRMDNF [17], CARN [18], IMDN [20], RFDN [49], ShuffleMixer [21], RLFN [49], ESRT [31], LBNet [32], SAFMN [50], and NGswin [28].

#### 4.3.1. Quantitative Results Analysis

Table 4 presents the quantitative comparison of our proposed PSNLMN with a range of representative methods on four benchmark datasets under scale factors ×2, ×3, and ×4. PSNLMN demonstrates a considerable improvement in performance across all settings.

Scale ×2: On Set14, PSNLMN achieves a PSNR of 34.27 dB, outperforming the best baseline NGswin (33.79 dB) by +0.48 dB. On Urban100, SSIM improves from 0.9324 (NGswin) to 0.9447, a gain of +0.0123. PSNLMN also achieves the highest SSIM on all four datasets under this scale. Although the PSNR on Set5 (38.06 dB) and Urban100 (32.55 dB) is slightly lower than the best values (38.10 dB by BSRN and 32.58 dB by ESRT), PSNLMN surpasses them in SSIM by a clear margin, indicating improved structural similarity and visual fidelity.

Scale ×3: PSNLMN achieves substantial improvements in SSIM across all datasets. For example, on Set14, SSIM increases from 0.8456 (NGswin) to 0.8689 and, on BSD100, from 0.8078 to 0.8266, representing gains of +0.0233 and +0.0188, respectively. On Urban100, SSIM improves from 0.8603 to 0.8740 (+0.0137), and PSNR from 28.52 dB to 28.71 dB (+0.19 dB). While PSNR improvements on Set5 and BSD100 are relatively modest, the SSIM consistently ranks first, highlighting better texture and detail preservation.

Scale ×4: At this scale, PSNLMN consistently achieves the highest SSIM across all datasets. On Set5, the SSIM improves from 0.8966 (BSRN) to 0.9059 and, on Urban100, from 0.7963 (NGswin) to 0.8133, representing a notable improvement of 0.017. On BSD100, the SSIM increases by 0.0201, reaching 0.7597. Although the PSNR on Set5 (32.33 dB) and Set14 (28.74 dB) is slightly lower than that of the best-performing methods by 0.02–0.04 dB, the consistently superior SSIM indicates enhanced structural fidelity and perceptual quality.

In summary, PSNLMN delivers significant SSIM improvements across all datasets and scales, especially on texture-rich scenes like Set14 and Urban100. Although PSNR is not always the highest, it remains competitive, and the consistent SSIM superiority indicates better structural fidelity and perceptual quality of the reconstructed images. These results validate the effectiveness of our approach in enhancing both objective metrics and visual realism.

#### 4.3.2. Qualitative Results Analysis

**Figure 8:**
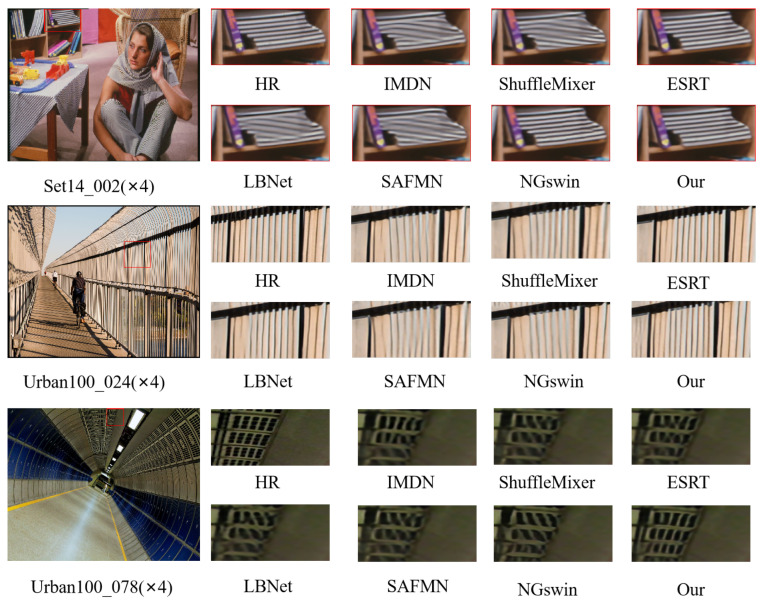
Visual comparison of ×4 SR methods on the benchmark test set (The area used for comparison is marked with a red box).

Figure 8 shows a visual comparison of reconstructed images by different methods, all under the challenging ×4 upscaling setting. We focus on the ×4 scale because it presents the greatest difficulty among all tested magnification levels. At this scale, artifacts and detail loss are more apparent, making it a better indicator of a model’s ability to recover fine structures. Therefore, it serves as a representative case for evaluating the perceptual quality and detail preservation of super-resolution algorithms.

In the first example, both PSNLMN and NGswin accurately reconstruct the striped texture on the bookshelf, maintaining clear and continuous lines. In contrast, methods such as IMDM and ShuffleMixer produce blurred or broken stripe patterns, failing to preserve the original structure. In the second example, PSNLMN recovers the dense line patterns of the bridge with higher precision, while the other methods show visible distortion or smearing. In the third example, which involves a tunnel ceiling with grid-like structures, PSNLMN preserves the regularity and sharpness of the grid, whereas competing methods tend to distort the geometry or lose detail. These visual results further demonstrate the superior ability of PSNLMN to retain fine-grained textures and structural integrity, especially in complex scenes with rich high-frequency content.

## 5. Conclusions

In this paper, we propose a novel architecture for SISR. The architecture integrates two key components: (1) a PSNLM grounded in NLM theory and multi-head self-attention mechanisms; and (2) an ADMFB to enhance multi-scale feature representation capabilities. This hybrid architecture leverages the complementary properties of CNNs and Transformers, aiming to mitigate their respective limitations. Specifically, the PSNLM module leverages a pixel-level self-attention mechanism to explicitly model long-range dependencies and non-local similarities. It reformulates the conventional NLM framework within a learnable self-attention paradigm, which enables dynamic weighting of image pixels based on domain patches with similar scenarios within the image. This mechanism helps to recover repetitive textures and long-range structures, whereas standard CNNs often miss due to limited receptive fields. Concurrently, the ADMFB employs a dual-path attention mechanism within a hierarchical feature fusion strategy. It adaptively fuses multi-scale features extracted from parallel branches possessing varying receptive fields. This design not only enriches feature diversity but also optimizes the trade-off between computational efficiency and multi-scale representation capabilities.

By cascading the PSNLM and ADMFB modules within an overarching CNN framework, our proposed architecture synergistically leverages both efficient local feature processing and comprehensive global and multi-scale feature representation. The experimental validation on benchmark datasets (Set5, Set14, BSD100, and Urban100) demonstrates that the proposed architecture outperforms most state-of-the-art methods across three distinct SISR tasks, measured by quantitative metrics (PSNR/SSIM) and qualitative visual effects, yielding more natural-looking reconstructions.

The practical applicability of the proposed architecture is particularly relevant for resource-constrained sensor systems where hardware upgrades are often infeasible, yet high-resolution image reconstruction remains critical for downstream applications such as medical diagnosis, autonomous navigation, and remote sensing analysis. By combining local and global modeling, the framework offers a balance between accuracy and efficiency for real-world super-resolution tasks.

However, the proposed framework still has several limitations. First, the incorporation of attention-based modules increases computational overhead compared to lightweight architectures, which may hinder its real-time deployment on edge devices. Second, its robustness under complex degradation scenarios remains to be further validated.

In future work, we plan to extend the proposed architecture to handle more complex and realistic degradation models. We also aim to investigate its generalization ability across different domains and image restoration tasks.

## Figures and Tables

**Table 1:** Results of ablation study on PSNLM. The best results are highlighted in bold.

Model	RB	NLMB_PL	NLMB_P	Set5		Set14		BSD100		Urban100	
				PSNR	SSIM	PSNR	SSIM	PSNR	SSIM	PSNR	SSIM
Base_Model	✓	✗	✗	31.607	0.8984	27.942	0.8029	27.419	0.7502	25.670	0.7870
Model_PSNLM_L	✗	✓	✗	31.929	0.9024	28.217	0.8086	27.561	0.7549	26.098	0.7994
Model_PSNLM	✗	✗	✓	**32.021**	**0.9036**	**28.250**	**0.8098**	**27.613**	**0.7563**	**26.161**	**0.8021**

**Table 2:** Results of ablation study on ADMFB. The best results are highlighted in bold.

Model	RB	Wide-Focus	Path1	Path2	Set5		Set14		BSD100		Urban100	
					PSNR	SSIM	PSNR	SSIM	PSNR	SSIM	PSNR	SSIM
Base_Model_WF	✓	✓	✗	✗	31.794	0.9010	28.030	0.8050	27.406	0.7535	25.803	0.7947
Base_Model_P1	✓	✗	✓	✗	31.835	0.9011	28.102	0.8065	27.476	0.7539	25.897	0.7951
Base_Model_P2	✓	✗	✗	✓	31.898	0.9014	28.175	0.8068	27.515	0.7541	25.937	0.7956
Model_ADMFB	✓	✗	✓	✓	**31.962**	**0.9026**	**28.213**	**0.8077**	**27.598**	**0.7560**	**26.084**	**0.7989**

**Table 3:** Results of ablation study on combined modules. The best results are highlighted in bold.

Model	Num_branch	NLMB_P	Path1	Path2	Set5		Set14		BSD100		Urban100	
					PSNR	SSIM	PSNR	SSIM	PSNR	SSIM	PSNR	SSIM
Model_PSNLM	3	✓	✗	✗	32.021	0.9036	28.250	0.8098	27.613	0.7563	26.161	0.8021
Model_ADMFB	3	✗	✓	✓	31.962	0.9026	28.213	0.8077	27.598	0.7561	26.084	0.7989
PSNLMN_2	2	✓	✓	✓	32.024	0.9031	28.271	0.8088	27.573	0.7552	26.04	0.7986
PSNLMN_4	3	✓	✓	✓	32.132	0.9037	28.542	0.8108	27.643	0.7568	26.237	0.8029
PSNLMN_3	4	✓	✓	✓	**32.144**	**0.9039**	**28.572**	**0.8110**	**27.650**	**0.7571**	**26.243**	**0.8030**

**Table 4:** Quantitative comparison of image SR with other methods (PSNR/SSIM). The best and second-best results are highlighted using bold and underlined, respectively.

Method	Scale	Set5	Set14	BSD100	Urban100
		PSNR/SSIM	PSNR/SSIM	PSNR/SSIM	PSNR/SSIM
MemNet [15]	×2	37.78/0.9597	33.28/0.9142	32.08/0.8978	31.31/0.9195
SRMDNF [17]	×2	37.79/0.960	33.32/0.915	32.05/0.898	31.33/0.920
CARN [18]	×2	37.76/0.9590	33.52/0.9166	32.09/0.8978	31.92/0.9256
IMDN [20]	×2	38.00/0.9605	33.63/0.9177	32.19/0.8996	32.17/0.9283
RFDN-L [49]	×2	38.08/0.9606	33.67/0.9190	32.18/0.8996	32.24/0.9290
ShuffleMixer [21]	×2	38.01/0.9606	33.63/0.9180	32.17/0.8995	31.89/0.9257
ESRT [31]	×2	38.03/0.9600	33.75/0.9184	32.25/0.9001	32.58/0.9318
LBNet [32]	×2	38.05/0.9607	33.65/0.9177	32.16/0.8994	32.30/0.9291
SAFMN [50]	×2	38.00/0.9605	33.54/0.9177	32.16/0.8995	31.84/0.9256
BSRN [51]	×2	**38.10**/0.9610	33.74/0.9193	32.24/0.9006	32.34/0.9303
NGswin [28]	×2	38.05/0.9610	33.79/0.9199	32.27/0.9008	32.53/0.9324
PSNLMN(our)	×2	38.06/**0.9654**	**34.27/0.9349**	**32.31/0.9119**	32.55/**0.9447**
MemNet [15]	×3	34.09/0.9248	30.00/0.8350	28.96/0.8001	27.56/0.8376
SRMDNF [17]	×3	34.12/0.925	30.04/0.837	28.97/0.803	27.57/0.840
CARN [18]	×3	34.29/0.9255	30.29/0.8407	29.06/0.8034	28.06/0.8493
IMDN [20]	×3	34.36/0.9270	30.32/0.8417	29.09/0.8046	28.17/0.8519
RFDN-L [49]	×3	34.47/0.9280	30.35/0.8421	29.11/0.8053	28.32/0.8547
ShuffleMixer [21]	×3	34.40/0.9272	30.37/0.8423	29.12/0.8051	28.08/0.8498
ESRT [31]	×3	34.42/0.9268	30.43/0.8433	29.15/0.8063	28.46/0.8574
LBNet [32]	×3	34.47/0.9277	30.38/0.8417	29.13/0.8061	28.42/0.8559
SAFMN [50]	×3	34.34/0.9267	30.33/0.8418	29.08/0.8048	27.95/0.8474
BSRN [51]	×3	34.46/0.9277	30.47/0.8449	29.18/0.8068	28.39/0.8567
NGswin [28]	×3	34.52/0.9282	30.53/0.8456	29.19/0.8078	28.52/0.8603
PSNLMN(our)	×3	**34.56/0.9369**	**30.60/0.8689**	**29.23/0.8266**	**28.71/0.8740**
MemNet [15]	×4	31.74/0.8893	28.26/0.7723	27.40/0.7281	25.50/0.7630
SRMDNF [17]	×4	31.96/0.893	28.35/0.777	27.49/0.734	25.68/0.773
CARN [18]	×4	32.13/0.8937	28.60/0.7806	27.58/0.7349	26.07/0.7837
IMDN [20]	×4	32.21/0.8948	28.58/0.7811	27.56/0.7353	26.04/0.7838
RFDN-L [49]	×4	32.28/0.8957	28.61/0.7818	27.58/0.7363	26.20/0.7883
ShuffleMixer [21]	×4	32.21/0.8953	28.66/0.7827	27.61/0.7366	26.08/0.7835
ESRT [31]	×4	32.19/0.8947	28.69/0.7833	27.69/0.7379	26.39/0.7962
LBNet [32]	×4	32.29/0.8960	28.68/0.7832	27.62/0.7382	26.27/0.7906
SAFMN [50]	×4	32.18/0.8948	28.60/0.7813	27.58/0.7359	25.97/0.7809
BSRN [51]	×4	**32.35**/0.8966	28.73/0.7847	27.65/0.7387	26.27/0.7908
NGswin [28]	×4	32.33/0.8963	**28.78**/0.7859	27.66/0.7396	26.45/0.7963
PSNLMN(our)	×4	32.33/**0.9059**	28.74/**0.8132**	**27.70/0.7597**	**26.56/0.8133**

## Data Availability

The data used in this study are publicly available.
